# Enhancing MaaS user satisfaction through strategic marketing: The synergy of sustainability and service experience

**DOI:** 10.1371/journal.pone.0316753

**Published:** 2025-01-27

**Authors:** LiYan Bu, Hong Chen, WeiCheng Pan, HeSen Li

**Affiliations:** School of Art and Design, Shenyang Aerospace University, Shenyang, China; Tallinn University of Technology School of Engineering: Tallinna Tehnikaulikool Inseneriteaduskond, ESTONIA

## Abstract

As urbanization intensifies and the need for sustainable transportation grows, Mobility as a Service (MaaS) emerges as a promising solution to urban mobility challenges. This study seeks to explore the underlying mechanisms of MaaS from a sustainability perspective and to assess its impact on service experience and user satisfaction. Additionally, it examines how user satisfaction influences the broader adoption of MaaS. To address these objectives, relevant hypotheses were posited, and hypothetical models were constructed based on a comprehensive review of the literature. The interconnections among sustainability, service experience, and user satisfaction within MaaS were rigorously analyzed employing both a survey methodology and structural equation modeling for data analysis. The findings support five hypotheses, affirming that sustainability significantly influences the MaaS service experience, which in turn impacts user satisfaction. Furthermore, sustainability directly contributes to user satisfaction and is crucial for its enhancement. User satisfaction also positively affects the dissemination of MaaS services. Notably, the study identifies a critical mediating role of service experience in the utilization of MaaS, linking sustainability and user satisfaction. This research offers both theoretical insights and practical guidance for understanding the operational dynamics of MaaS and improving the user experience.

## 1. Introduction

Sustainable development has increasingly permeated various sectors, including transportation. Sustainable mobility focuses on the sustainability of transportation modes and their environmental, social, and economic impacts. Embracing sustainable mobility practices can diminish traffic congestion, mitigate environmental pollution, and enhance urban accessibility [1]. This paradigm shift in urban mobility has fostered the development of innovative models like shared and intelligent mobility. Mobility as a Service (MaaS), a prominent innovation in sustainable mobility, aims to offer a more convenient, efficient, and environmentally friendly travel alternative, addressing urban transportation challenges [2]. The advancement of information technology and the growing prominence of urban transport issues have catalyzed the global expansion of MaaS. Numerous cities have introduced MaaS platforms that consolidate various transport modes (e.g., public transport, shared bikes, taxis) into a unified service, thereby meeting the diverse travel needs of users [3]. Moreover, MaaS provides urban transport managers with enhanced data and tools to optimize transport resource allocation, boost operational efficiency, and ameliorate the transportation environment. Research indicates that MaaS not only alleviates traffic congestion and curtails carbon emissions but also fosters economic growth and improves the quality of life for residents [4-7]. Despite its significant potential, MaaS faces several challenges and barriers, such as issues related to data sharing, privacy protection, service quality, and reliability [8,9]. Thus, further investigation is required to delve into the service experience and user satisfaction associated with MaaS to promote its sustainable advancement.Globally, governments and international organizations are vigorously advocating for MaaS. The European Commission, for instance, has issued a White Paper on Future Mobility proposing numerous policy measures and action plans to support MaaS development and facilitate the transformation of the European transport system [10]. The sustainability of MaaS services and the improvement of the MaaS service experience have thus emerged as critical areas of research in the field.

This research delves into the intrinsic mechanisms linking the sustainability of Mobility as a Service (MaaS) with service experience and user satisfaction from a sustainable development standpoint, and examines how user satisfaction influences the further promotion of MaaS. The study aims to uncover the potential impacts of urban sustainable development on MaaS and elucidate the roles played by service experience and user satisfaction within this framework. These insights are intended to expose the operational dynamics behind MaaS, thereby providing both theoretical foundations and practical guidelines for future urban transportation planning and the enhancement of MaaS services.Initially, this study conducts a comprehensive analysis of the sustainable value inherent in MaaS services, assessing how the urban environment, society, and economic factors influence the MaaS service experience while pinpointing the critical determinants of this experience. Subsequently, the research investigates the interactive mechanisms between MaaS service experience and user satisfaction. Building on sustainable service research, the study finally proposes to explore future development trajectories for MaaS services, aiming to offer theoretical insights and policy recommendations for fostering a more intelligent and sustainable urban transport system.This research is designed to yield insights and practical guidance for urban transportation planning, specifically enhancing Mobility as a Service (MaaS). By conducting a systematic investigation of the sustainability, user satisfaction, and service experience associated with MaaS, the study aims to contribute to the advancement of urban transportation systems towards more innovative, convenient, and environmentally sustainable paradigms.

### 1.1 Background and hypothesis

Mobility as a Service (MaaS) is increasingly recognized as an effective solution to the transportation challenges exacerbated by urbanization. This service aims to consolidate various modes of transportation into a seamless travel experience for users, thereby addressing issues such as traffic congestion and environmental pollution. Globally, cities have begun implementing MaaS initiatives, with Helsinki’s “Whim” application—a platform that integrates public transportation, shared bicycles, taxis, and other modes—serving as a prominent example [11,12]. Similarly, in Shenzhen, China, the “Shenzhen Tong” app enhances urban mobility by amalgamating services like buses, subways, and shared bicycles [13].Despite its potential, MaaS encounters several obstacles, including divergent technology standards, undeveloped policy and regulatory environments, and slow shifts in user behavior. While initial successes have been observed in some urban areas, the global advancement of MaaS remains in its nascent stages [14,15]. This study examines the interrelations among sustainability, service experience, user satisfaction, and the promotion of MaaS. Through positing critical hypotheses and constructing a hypothetical model, the research aims to elucidate the fundamental mechanisms driving these dynamics.

#### Sustainability, service experience, and customer satisfaction.

The Mobility as a Service (MaaS) sector stands to gain significantly from integrating sustainability initiatives that improve the user experience. Research indicates that transparency in pricing and the adoption of fair pricing strategies are vital for cultivating trust and satisfaction among MaaS users [16]. These elements enhance the service experience by ensuring users clearly understand their fees and perceive the pricing strategies as equitable. Furthermore, promotional policies and reward programs on MaaS platforms can augment service usage and convenience, positively influencing the overall service experience. From an environmental standpoint, MaaS providers that offer options for green travel and low-carbon transportation cater to environmentally conscious users. Such practices not only contribute to reducing carbon emissions but also fulfill the demand for sustainable mobility, thus boosting user satisfaction and loyalty towards MaaS services [17,18]. Engaging in such eco-friendly options allows MaaS providers to actively participate in environmental conservation, thereby enhancing the user experience. On the social dimension, implementing sustainability initiatives like social responsibility programs, community engagement, and forming equitable partnerships can foster increased trust and goodwill toward MaaS providers [19,20]. These practices help build a healthier and more inclusive service ecosystem, improving user satisfaction and loyalty, and fostering a favorable brand image. Overall, MaaS aims to deliver a comprehensive travel solution that is not only convenient and affordable but also environmentally friendly and socially inclusive. Therefore, sustainability can enrich the MaaS user experience across three dimensions: economic, environmental, and social. Based on the above discussion, the following hypotheses can be formulated:

H1: Sustainability positively affects the MaaS service experience in terms of economic, environmental, and social aspects.

Understanding users’ perception of service experience encompasses multiple dimensions: product experience, outcome focus, critical moments, and inner peace. Product experience pertains to the users’ perceptions of the service’s functions, features, and performance. Outcome focus addresses the tangible results users achieve after utilizing the service. Critical moments highlight the significance of pivotal interactions within the service process that shape overall impressions. Inner peace involves the comfort and tranquility users experience during service usage [21]. In the context of Mobility as a Service (MaaS), however, security emerges as a paramount concern. Users express considerable anxiety regarding the safeguarding of their services, especially in areas such as transportation selection, transaction payments, and personal information management. In today’s information age, the protection of individual privacy and data security has become a societal imperative [22]. MaaS providers are obligated to ensure the protection of users’ personal information, the security of payment transactions, and compliance with safety standards in transport and service facilities [23,24]. A secure environment not only serves as a fundamental prerequisite for utilizing MaaS services but also as a crucial component of user satisfaction and trust. Recent studies further underscore the critical role of health and safety in enhancing the service experience [25,26]. Consequently, security should be regarded as a core indicator of service quality within the service experience framework. Prioritizing security measures is essential for MaaS providers to foster user satisfaction and trust, thereby enhancing the overall customer experience.Based on the above discussion, the following hypotheses can be formulated:

H2: MaaS service experience consists of five key aspects, namely product experience, outcome focus, critical moments, inner peace, and security.

The advent of Mobility as a Service (MaaS) has reshaped how individuals choose their travel modes, increasingly swayed by not only convenience and cost but also the environmental implications of their options. The role of sustainability in influencing user satisfaction is becoming more pronounced [27]. In transportation, studies have demonstrated that services providing sustainable travel options often garner positive user reviews, which in turn lead to enhanced user satisfaction [28]. Further research supports that users are more likely to opt for travel methods that minimize carbon emissions and promote environmental friendliness, underscoring the beneficial effects of sustainability on user satisfaction [29]. In the realm of MaaS, there is a clear preference among users for services that resonate with their environmental values. This alignment is critical, as the literature provides compelling evidence of how sustainability factors influence user satisfaction within MaaS. By integrating a thorough analysis and synthesis of the existing literature, this study hypothesizes that offering eco-friendly and low-carbon travel options not only meets the basic travel needs of users but also fosters their identification with environmental responsibility. This identification, in turn, bolsters their willingness to support sustainable practices, thereby enhancing their satisfaction with MaaS services.Based on this, the study further proposes the following hypotheses:

H3: Sustainability has a positive impact on MaaS user satisfaction.

#### The relationship consequences of the MaaS service experience.

User satisfaction is increasingly recognized as a critical outcome of the service experience, particularly within the Mobility as a Service (MaaS) context [30]. In this framework, the satisfaction of MaaS users is determined by their evaluation of the service experience, specifically the discrepancy between the actual service received and their prior expectations [31]. Although existing research confirms the significant impact of service experience on user satisfaction, the dynamics of this relationship are complex and not entirely direct. For example, the design, functionality, and usability of a MaaS platform are known to directly influence users’ perceptions of the service, which in turn shapes their overall satisfaction [32]. Additionally, the convenience and degree of personalization offered by the service are crucial determinants of user satisfaction [33]. Moreover, it is essential for MaaS platforms to resonate with the values of their users, as this alignment significantly enhances user satisfaction. When the MaaS service experience meets users’ values and expectations, they are more likely to be content with the service and regard it as a superior travel option [34]. Given the intricacies of these relationships, there is a compelling need for more comprehensive studies to further validate and elucidate these findings. Based on the discussion above, this study proposes the following hypothesis:

H4: The MaaS service experience positively impacts user satisfaction with MaaS.

User satisfaction is pivotal in advancing Mobility as a Service (MaaS) systems. Primarily, satisfied users are more likely to endorse MaaS services to others, thus enhancing its visibility and recognition. This endorsement facilitates wider promotion and outreach, as evidenced by recent studies [35,36]. Additionally, users who are content with MaaS are more inclined to persist in using the services and to explore new features. Such behaviors disseminate positive information to others, thereby accelerating the promotion and outreach of MaaS [37]. Furthermore, satisfied users tend to be more engaged in the iterative improvement and development of MaaS services. By contributing feedback, participating in surveys, or engaging in community events, these users significantly amplify the visibility and promotional impact of the service [38]. Lastly, content users are more likely to become loyal customers. This loyalty fosters a sustained attachment to the MaaS brand and attracts new users through word-of-mouth, further bolstering the system’s promotion [39]. In conclusion, user satisfaction contributes positively to the promotion of MaaS through various channels such as word-of-mouth, behavioral intentions, engagement, and loyalty. These dynamics not only drive improvements within the system but also support its broader adoption, thus contributing to the sustainable development of urban transportation. Therefore, this study proposes the following hypothesis:

H5: User satisfaction with MaaS positively impacts its promotion.

**Fig 1:**
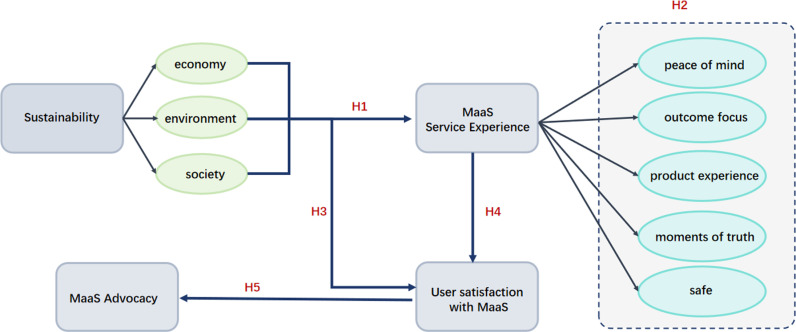
Hypothetical model.

This study presents a hypothetical model, constructed upon the five hypotheses delineated previously. The model is designed to investigate the interrelationships among sustainability, service experience, user satisfaction, and the promotion of Mobility as a Service (MaaS). It offers a comprehensive framework aimed at elucidating how these variables collectively impact the effectiveness and expansion of MaaS systems. This figure shows the study’s hypothetical model of sustainability, MaaS service aspects, user satisfaction, and advocacy. It details how sustainability impacts them directly/indirectly and how user satisfaction affects MaaS promotion (See Fig 1).

## 2. Methods

This study defines MaaS as a platform that integrates various transportation modes into a single, seamless service, including public transit, bike-sharing, taxis, and ride-hailing services. A brief explanation accompanied each question related to MaaS to ensure participants’ understanding. In order to address the research gap in Mobility as a Service (MaaS), this study enhanced and expanded the Experience Quality (EXQ) scale through a comprehensive literature review, expert interviews, and an initial user survey. A safety dimension was also integrated to comprehensively encompass the unique features of MaaS, as outlined in Table 4. The EXQ scale is renowned for its detailed structure and validated applicability in service experience research, effectively measuring user experience quality across dimensions such as convenience, safety, and reliability [40]. Although initially developed for contractual service experiences, its multidimensional approach is well-suited to capturing the complex experience factors inherent in MaaS. Its successful application in various fields underscores its flexibility and reliability, highlighting its capability to reflect the multifaceted nature of MaaS service experiences. This study enhances and extends the application of the EXQ scale within the urban transportation context, ensuring its relevance and validity in MaaS research.

Structural Equation Modeling (SEM) is a statistical technique used to construct and validate complex models of relationships between observed variables. It combines measurement and causal relationship models, enabling the assessment of direct and indirect effects among multiple variables and their causal relationships [41].

### 2.1 Sample selection and data collection

This study employed the Experience Quality (EXQ) scale to investigate the service experience of Mobility as a Service (MaaS), integrating a new “safety” dimension derived from a comprehensive literature review and user interviews. See Table 1 for details. A questionnaire was meticulously designed with three pertinent questions per dimension, utilizing a 5-point Likert scale to capture user attitudes and perceptions precisely. This scale effectively measures subjective variables, ensuring the data’s validity and reliability. The questionnaire covered crucial aspects of sustainability, MaaS user satisfaction, and MaaS advocacy. Each section included detailed descriptions, and a reliability analysis was performed to ensure the data’s robustness.

**Table 1:** Sources of Literature.

Variables	References	Items
**MaaS Service Experience**	Klaus and Maklan (2012) [63]	15
Peace of mind		3
Outcome focus		3
Product experience		3
Moments of truth		3
Safe	Singleton & Wang (2014) [64]	3
**Sustainability**	Grunkemeyer & Moss (2020) [62]	9
Economic sustainability		3
Environmental sustainability		3
Society sustainability		3
**User satisfaction with MaaS**	Al‐Maskari & Sanderson (2010) [65]	3
**MaaS Advocacy**	Stokburger et al. (2012) [66]	3

This survey distributed 450 questionnaires. The study eliminated 33 invalid questionnaires: 18 were excluded due to incomplete input and 15 due to inconsistent responses. Consequently, 417 valid questionnaires were retained, yielding a % effective response rate of 92.67%. Detailed breakdowns can be found in Table 2. Detailed breakdowns can be found in Table 2. The alpha coefficient (α) is utilized to assess the internal consistency reliability of a scale, with higher α values signifying improved scale reliability. This implies greater consistency among the items within the questionnaire [42]. In this study, the questionnaire demonstrated high stability, with reliability coefficients for its various dimensions and variables ranging from 0.768 to 0.852, suggesting a respectable level of credibility. Further details are presented in Table 4.

**Table 2:** Sample Characteristics - Gender, Age, Education Level, Usage Frequency.

	Categories	Frequency (times)	Percentage (%)
Gender	male	188	45.1
	female	229	54.9
Age	Under 18 years old	23	5.5
	18–25	82	19.7
	26–40	144	34.5
	41–60	89	21.3
	Over 60 years old	79	18.9
Education	Junior high school and below	25	6
	high school	55	13.2
	university	233	55.9
	Graduate or above	104	24.9
MaaS service usage	never used	30	7.2
	1 to 5 times a year	255	61.2
	More than 5 times a year	132	31.6

## 3. Results and analysis

### 3.1 Reliability and validity analyses

Structural Equation Modeling (SEM) was used for Confirmatory Factor Analysis (CFA) on the MaaS service experience scale and sustainability models. The analysis was performed using SPSS version 26.0 and AMOS version 26.0 software. The fit indices are as follows: ^2^/df = 1.570; GFI = 0.961; AGFI = 0.945; IFI = 0.982; TLI = 0.977; CFI = 0.982; RMSEA = 0.037. The model’s fit indices met the required standards, making it suitable for further analysis—the validity of the MaaS service experience model dimensions and corresponding questionnaire items. Convergent validity was tested by assessing the construct reliability (CR >  0.7) and the average variance extracted (AVE >  0.5). The AVE square root for each model dimension was more significant than the correlation coefficients between the dimensions, indicating that the scale has good discriminant validity. See Table 3 for more information.

**Table 3:** Discriminant Validity Analysis of MaaS User Experience.

	Safe	Moments of truth	Product experience	Outcome focus	Peace of mind
Safe	0.781				
Moments of truth	0.424	0.788			
Product experience	0.397	0.511	0.758		
Outcome focus	0.482	0.619	0.580	0.724	
Peace of mind	0.418	0.538	0.504	0.611	0.788

### 3.2 Data analyses

The study employed multiple validation tests to ascertain the robustness of the model and ensure that the refinement process maintained the scale’s reliability. These tests involved evaluating the average variance extracted (AVE >  0.5), construct reliability (CR >  0.7), and Cronbach’s alpha (α >  0.7), each assessing the internal consistency of the model. Additionally, the study examined both convergent and discriminant validity. Convergent validity was confirmed through significant and high correlation estimates among the dimensions of the constructs. Discriminant validity was assessed using confidence intervals and AVE tests, with the results affirming its presence. Detailed results and further elaborations are available in Table 4.

**Table 4:** Results After the Experimental Refinement Process.

Items	CR	C.R.
**MaaS Service Experience (CR:0.841 AVE:0.517)**		
**peace of mind (CR:0.831; AVE:0.621;** **α=0.830)**		
PM1: During the booking and use of transportation services, I am distinctly aware of my responsibilities.	0.848	
PM2: The procedures involved in booking and utilizing transportation services are straightforward and the journey proceeds smoothly.	0.758	15.793
PM3: The recommendations provided during the booking and use of transportation services are objective and appropriate.	0.756	15.753
**moments of truth (CR:0.831; AVE:0.621;** **α=0.831)**		
MT1: Personnel inquire about my needs during the booking and use of transportation services.	0.796	
MT2: I am consistently updated with all relevant information during the booking and use of transportation services.	0.798	15.866
MT3: When issues arise during the booking and use of transportation services, personnel and software can address them promptly and effectively.	0.77	15.398
**outcome focus (CR:0.767; AVE:0.524;** **α=0.768)**		
OF1: The process of booking and utilizing transportation services is as seamless as I anticipated.	0.700	
OF2: I feel more confident in the booking and use of transportation services compared to previous travel experiences.	0.682	11.786
OF3: Personnel and software effectively address my concerns, facilitating a smooth journey.	0.784	12.925
**product experience (CR:0.802; AVE:0.575;** **α=0.801)**		
PE1: The transportation service provides diverse recommendations, such as cultural sites, leisure spots, and dining locations.	0.749	
PE2: I have the option to choose from various shared transportation modes during use.	0.796	14.053
PE3: Personnel provide assistance throughout the booking and use of transportation services.	0.728	13.281
**safe (CR:0.825; AVE:0.611;** **α=0.824)**		
SA1: The transportation service ensures a secure environment and implements relevant safety measures, enhancing your sense of safety.	0.792	
SA2: Fellow passengers exhibit high levels of civic behavior during travel.	0.760	14.714
SA3: The transportation tools provide comfortable and clean riding conditions.	0.792	15.140
**Sustainablility (CR:0.769 AVE:0.527)**		
**economy (CR:0.854; AVE:0.662;** **α=0.852)**		
EC1: The transportation service contributes positively to urban economic development.	0.769	
EC2: MaaS receives commercial investment in transportation infrastructure.	0.878	17.079
EC3: MaaS creates multiple employment opportunities for residents without discrimination.	0.789	15.976
**environment (CR:0.840; AVE:0.636;** **α=0.839)**		
EN1: Utilizing the transportation service reduces environmental impact.	0.810	
EN2: Discarded transportation tools are recyclable.	0.788	15.946
EN3: Compared to other cities, MaaS helps the local city maintain lower pollution levels.	0.794	16.039
**society (CR:0.822; AVE:0.606;** **α=0.821)**		
SO1: MaaS enhances the city’s cultural image.	0.772	
SO2: MaaS promotes cultural and social development in the local city.	0.756	14.467
SO3: MaaS provides infrastructure accommodating disabled individuals.	0.806	15.128
**User satisfaction with MaaS (CR:0.840; AVE:0.637;** **α=0.840)**		
US1: I am satisfied with all services provided during my journey.	0.785	
US2: I find the pricing of the provided services satisfactory.	0.830	16.315
US3: My overall experience of the journey is positive.	0.778	15.555
**MaaS Advocacy (CR:0.834; AVE:0.626;** **α=0.833)**		
MA1: I inform others about MaaS (Mobility as a Service).	0.822	
MA2: I actively recommend MaaS (Mobility as a Service) to others.	0.781	15.516
MA3: I encourage family and friends to try MaaS (Mobility as a Service).	0.770	15.348
Fit of the model: c^2^/df = 1.187; GFI = 0.938; AGFI = 0.921; IFI = 0.988; TLI = 0.986; CFI = 0.988; RMSEA = 0.021		

Note: AVE  = average variance extracted; CR  = composite reliability;

* p < 0.001.

The experiment focused on analyzing the discriminant validity of the overall scale. The results indicated that the square root of the Average Variance Extracted (AVE) for each dimension exceeded the correlation coefficients between the dimensions, demonstrating robust discriminant validity of the scale. Further details can be found in Table 5.

**Table 5:** Overall Scale Discriminant Validity Analysis.

	1	2	3	4	5	6	7	8	9	10
User satisfaction with MaaS	0.798									
MaaS advocacy	0.251	0.813								
Economy	0.317	0.376	0.791							
Environment	0.317	0.485	0.357	0.797						
Society	0.360	0.523	0.400	0.562	0.778					
Peace of mind	0.438	0.181	0.330	0.180	0.150	0.788				
Outcome focus	0.468	0.247	0.312	0.210	0.170	0.620	0.724			
Product experience	0.362	0.306	0.321	0.193	0.184	0.474	0.569	0.758		
Moments of truth	0.491	0.204	0.261	0.243	0.151	0.541	0.640	0.506	0.788	
Safe	0.495	0.178	0.318	0.224	0.210	0.434	0.447	0.484	0.384	0.781

**Fig 2:**
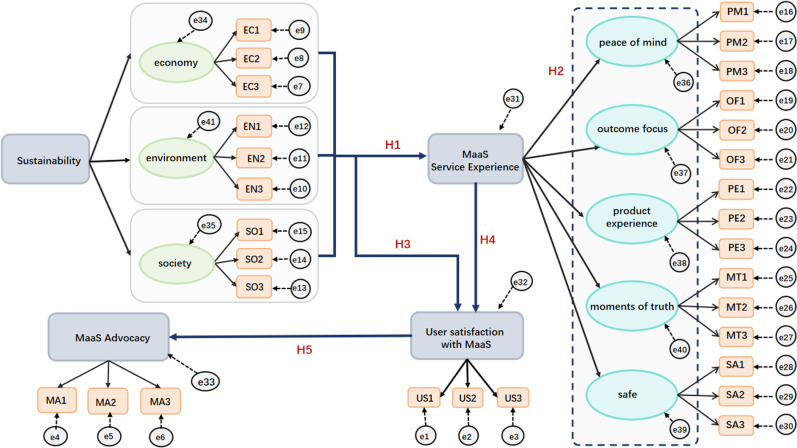
SEM step diagram.

**Fig 3:**
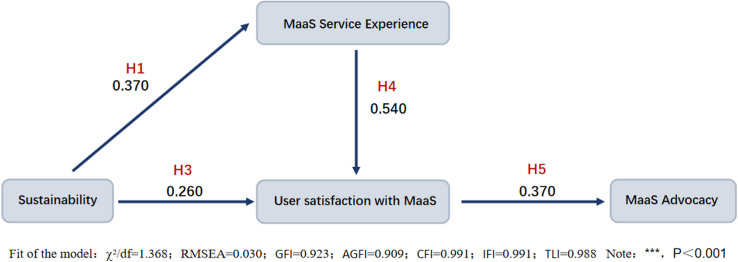
Results of the structural model.

The experiment employed SPSS 26.0 and AMOS 26.0 software for hypothesis testing within the model, facilitating the simultaneous examination of interrelationships among variables. Figs 2 and 3 depict the model steps and results, respectively. Fig 2 depicts the SEM implementation steps in MaaS research, beginning with variable identification (sustainability, service exp., user sat., MaaS advocacy), followed by data processing/analysis and model refinement/validation to determine variable relationships. Fig 3 shows the structural model results, with estimated path coefficients and significance levels among the variables. The results confirm hypothesized relationships and reveal the direct/indirect effects, emphasizing the importance of sustainability and service experience in user satisfaction and MaaS promotion.

According to the parameter estimates (λ) derived from the model results, sustainability exerts a positive and significant impact on the Mobility as a Service (MaaS) experience from three dimensions (H1: λ =  0.370). However, its direct effect on user satisfaction is less pronounced (H3: λ =  0.260). In contrast, the MaaS service experience significantly positively affects user satisfaction (H4: λ =  0.540), and this satisfaction, in turn, positively influences the promotion of MaaS (H5: λ =  0.370). These findings underscore the pivotal role of service experience in enhancing user satisfaction and promoting MaaS, indicating strategic areas for potential improvement to boost overall system effectiveness.

The research conducted a comprehensive analysis of the Structural Equation Modeling (SEM) pathways within the overall model. This analysis included deriving the path coefficient values and Critical Ratio (C.R.) values, which elucidate the extent of influence and relationships between variables. A regression coefficient is considered statistically significant if the C.R. value is 1.96 or higher. It can refer to Table 6 for further details.

**Table 6:** Total Effects.

Regression Pathways	β	b	S.E.	C.R.
MaaS service experience←Sustainability	0.373	0.551	0.111	4.981
user satisfaction with MaaS ← Sustainability	0.256	0.451	0.113	3.988
user satisfaction with MaaS ← MaaS service experience	0.541	0.645	0.083	7.733
MaaS advocacy←user satisfaction with MaaS	0.365	0.293	0.047	6.252

Note: ***, P <  0.001; b: unstandardized coefficients; β: standardized factor coefficients.

The key findings from the study are as follows

Sustainability and MaaS Service Experience: Sustainability has a demonstrably positive impact on the Mobility as a Service (MaaS) service experience. The standardized path coefficient for this relationship is 0.373, with a C.R. value of 4.981, significantly exceeding the threshold of 1.96. The corresponding p-value is less than 0.001, substantiating the validity of the hypothesis.

Sustainability and User Satisfaction with MaaS: The analysis shows that sustainability positively influences user satisfaction with MaaS, with a standardized path coefficient of 0.256. The C.R. value is 3.988, also surpassing the threshold of 1.96. The corresponding p-value is less than 0.001, further confirming the hypothesis’s validity.

MaaS Service Experience and User Satisfaction: The MaaS service experience significantly positively impacts user satisfaction, as indicated by a standardized path coefficient of 0.541. The C.R. value for this effect is 7.733, well above the threshold of 1.96. The corresponding p-value is less than 0.001, verifying the hypothesis’s validity.

User Satisfaction and MaaS Advocacy: User satisfaction with MaaS positively impacts MaaS advocacy. The standardized path coefficient is 0.365, with a C.R. value of 6.252, exceeding the significance threshold. The corresponding p-value is less than 0.001, affirming the hypothesis’s validity.

Further research is warranted to ascertain the presence of any mediating effects within the significant pathways identified in the model. The study utilized the Bootstrap method in AMOS 26.0, employing 5000 repetitions, setting a confidence interval standard at 95%, and applying bias correction for robust testing. For comprehensive results, please see Table 7. The bias-corrected Bootstrap confidence intervals for the mediated pathways indicate significant mediating effects, as follows:

**Table 7:** Mediating Effects.

Regression Pathways	Effect Size	SE	95% Lower Limit	95% Upper Limit
Sustainability→MaaS service experience→user satisfaction with MaaS	0.202	0.045	0.124	0.302
Sustainability→user satisfaction with MaaS → MaaS advocacy	0.198	0.042	0.123	0.284
MaaS service experience→user satisfaction with MaaS → MaaS advocacy	0.093	0.030	0.043	0.160

Sustainability to MaaS Service Experience to User Satisfaction with MaaS: The confidence interval, ranging from 0.124 to 0.302, does not include zero, signifying a substantial mediating effect.

Sustainability to User Satisfaction with MaaS to MaaS Advocacy: The confidence interval, extending from 0.123 to 0.284, does not encompass zero, thus confirming the presence of a mediating effect.

MaaS Service Experience to User Satisfaction with MaaS to MaaS Advocacy: The confidence interval, spanning from 0.043 to 0.160, excludes zero, indicating a significant mediating effect.

## 4. Discussion

This study explores the interrelationships among sustainability, Mobility as a Service (MaaS) service experience, and user satisfaction, alongside the influence of MaaS user satisfaction on the promotion of MaaS. The proposed hypotheses are analyzed with the following results:

Hypothesis 1: Sustainability positively affects the MaaS service experience across economic, environmental, and social dimensions. Sustainability is recognized as a pivotal element of modern urban transportation frameworks, particularly within the context of MaaS platforms [43]. Vitetta emphasizes the integration of sustainable objectives into MaaS, scrutinizing their impact on the design and service experience [44]. Economically, MaaS offers cost-effective and convenient transportation solutions that enhance service experience by reducing costs and improving convenience [45]. Environmentally, it supports shared and eco-friendly transportation modes, such as public transit, biking, and electric vehicles, aligning with increasing user concerns about climate change and pollution [46]. Socially, MaaS enhances inclusivity and accessibility by providing transportation alternatives for various user groups, including those with mobility restrictions or without private vehicle access [47]. By integrating these economic, environmental, and social aspects, aligning MaaS services with sustainable development goals significantly improves the user experience. Furthermore, applying sustainability metrics in evaluating MaaS services can yield insights into user preferences and satisfaction levels, filling a research gap regarding the impact of sustainability on the MaaS experience. This hypothesis lays a theoretical foundation for further investigation into how specific sustainability initiatives affect the MaaS user experience. It is imperative for MaaS service providers and policymakers to prioritize sustainability initiatives, as these efforts are crucial for enhancing the appeal and efficacy of MaaS platforms. This research will aid in optimizing service strategies, improving user experiences, and advancing the realization of sustainable urban mobility.

Hypothesis 2: The MaaS (Mobility as a Service) service experience is comprised of five key aspects: product experience, outcome focus, critical moments, inner peace, and security.The MaaS service experience is inherently multifaceted, encapsulating a range of dimensions that collectively influence users’ perceptions and satisfaction with MaaS services. Product experience encompasses the quality, functionality, and usability of MaaS platforms, including user interfaces, booking processes, and payment systems, which are crucial for user engagement [48]. Outcome focus relates to the extent to which MaaS services fulfill users’ transportation needs and preferences, such as route efficiency, travel time savings, and flexibility in mode selection [49]. Critical moments during the user journey, including service interruptions or transitions between modes, play a pivotal role in shaping overall satisfaction [50]. Inner peace addresses the emotional well-being of users while utilizing MaaS, focusing on comfort, convenience, and confidence in the system’s reliability and predictability [51]. Lastly, security is essential, covering aspects of physical safety during travel, as well as data security and privacy protection, which are fundamental in building user trust and confidence [52]. Understanding these components in depth provides both a theoretical basis and practical insights for enhancing the quality of MaaS services and customer satisfaction. MaaS service providers are advised to prioritize user-centered design and seamless integration of these service experiences to ensure user satisfaction.

Hypothesis 3: Sustainability positively impacts user satisfaction within Mobility as a Service (MaaS). The sustainability of MaaS significantly enhances user satisfaction across various dimensions. Economically, MaaS provides cost-effective and flexible transportation options, which not only save users money but also add convenience, appealing broadly to consumers [53]. Environmentally, sustainability initiatives resonate with environmentally conscious users, thereby increasing their satisfaction with the MaaS platform. Socially, MaaS services promote inclusivity and accessibility by offering diverse transportation solutions for user groups with different needs, including those with mobility restrictions and marginalized communities [54]. Moreover, the social sustainability of MaaS services can be enhanced through effective partnerships with communities and stakeholders, fostering broader societal benefits [55]. Aligning MaaS services with sustainable development goals not only meets users’ economic, environmental, and social preferences but also substantially enhances their overall satisfaction. This hypothesis underscores the profound influence of sustainability on MaaS user satisfaction and delves into the mechanisms through which sustainability factors affect user contentment. By examining user attitudes towards sustainability and their connection to satisfaction, this study provides a deeper understanding of user needs and expectations. It also introduces fresh perspectives for future research, aiding MaaS service providers in crafting more effective service strategies. To boost user satisfaction and loyalty, it is crucial for MaaS providers to transparently communicate their sustainability initiatives to their users. Consequently, sustainability considerations are deemed essential in the development and enhancement of MaaS services.

Hypothesis 4: The MaaS service experience positively impacts user satisfaction with Mobility as a Service (MaaS). User satisfaction with MaaS is intricately linked to the quality of the service experience offered by the platform. Industry experts advocate that MaaS services should focus on enhancing user satisfaction by providing a user-friendly interface, streamlined booking processes, and reliable transportation options [56]. Through investments in technological advancements and innovative features, MaaS platforms can enhance their functionality and user-friendliness, thereby boosting user satisfaction. Positive service experiences characterized by convenience, efficiency, and accessibility are known to foster user engagement and loyalty towards MaaS platforms. Additionally, fulfilling user expectations through timely arrivals, accurate route information, and effective customer support significantly contributes to overall satisfaction with MaaS services. It is essential for MaaS providers to prioritize user-centered design and continuously assess user feedback and preferences to refine and enhance their services continually. This strategic focus on improving the service experience can lead to higher levels of user satisfaction. This hypothesis provides vital theoretical backing for the relationship between the quality of MaaS service experience and user satisfaction. By further exploring this relationship, valuable insights can be offered for urban planning and traffic management, aiming to improve the efficiency and sustainability of urban transportation systems.

Hypothesis 5: User satisfaction with Mobility as a Service (MaaS) positively impacts its promotion. Empirical research indicates that satisfied MaaS users are more likely to recommend and promote the service to others, thereby facilitating broader adoption of the platform [57]. User satisfaction is a pivotal factor in the promotional behaviors of users in the service industry. Reliable transportation services, cost savings, and environmental benefits are significant motivators for users to share their positive experiences with peers, family, and colleagues [58,59]. Encouraging participation in referral programs and offering incentives can further leverage satisfied users to promote the MaaS platform within their networks. Furthermore, satisfied users are likely to engage with MaaS providers via feedback mechanisms, social media platforms, and community forums, thus amplifying positive word-of-mouth and enhancing promotional efforts [60,61]. By prioritizing user satisfaction and fostering a user-centered culture, MaaS providers can treat satisfied users as crucial promoters and ambassadors of the service. Implementing personalized communication and engagement programs can help establish and strengthen relationships with satisfied users, thereby boosting promotion and loyalty towards MaaS services. This hypothesis underscores substantial research and practical implications, offering valuable insights for the design and promotional strategies of MaaS systems, which contribute to the sustainable development of urban transportation.

Based on the insights gained from the five hypotheses investigated, this research proposes four strategic recommendations to enhance the sustainable development of MaaS:

Urban Government Policy Support: Governments should establish a sustainable transportation policy framework that fosters the growth of MaaS. This can involve integrating public transport with shared mobility services while reducing reliance on private cars. Encouraging the use of MaaS over private vehicles can be facilitated through preferential policies such as subsidies or discounts specifically tailored for MaaS users. Additionally, urban planning and infrastructure development should prioritize MaaS needs, including the establishment of dedicated parking spaces or charging stations and optimizing traffic mobility.

Service Experience Enhancement for MaaS Providers: MaaS platform providers should concentrate on continuously improving the user interface and functionality of their platforms. This improvement can be achieved by harnessing new technologies like Artificial Intelligence and Data Analytics to personalize and optimize service experiences. Providers should offer a diverse array of user-friendly travel options that cater to various user groups, including individuals with disabilities or families, to enhance accessibility and inclusivity.

Collaboration with Communities and Stakeholders: It is crucial to establish and maintain effective communication channels, such as public forums and workshops, with communities and stakeholders. This collaboration will enable local governments and business entities to address community-specific transportation issues and promote sustainable transportation solutions together. Prioritizing social inclusiveness and ensuring that MaaS services are accessible and equitable for individuals with diverse physical abilities and from various socioeconomic backgrounds is essential.

Establishing a User Feedback Mechanism and Social Media Management: MaaS providers should offer diversified feedback channels, including in-app functions, online surveys, and customer service hotlines, enabling users to easily submit their opinions and suggestions. Prompt and active responses to user feedback, resolving issues efficiently, and maintaining a reputable service standard are critical. Additionally, utilizing social media platforms to engage with users actively, disseminate information about MaaS services, and increase public awareness and participation in sustainable travel is recommended.

## 5. Conclusions

The study bases its analysis on a comprehensive review of existing literature, along with data collected from questionnaire surveys. Structural equation modeling was used to test the relationships between sustainability, service experience, and user satisfaction in MaaS.The findings confirm that sustainability significantly enhances the MaaS service experience, which subsequently boosts user satisfaction. Direct impacts of sustainability on user satisfaction were also observed, underscoring its importance in improving user experiences. Additionally, a positive relationship between user satisfaction and the promotion of MaaS was established, with service experience acting as a key mediator between sustainability and user satisfaction.These results provide crucial insights into the mechanisms by which MaaS services can transform urban transportation. The mediating role of service experience links sustainability directly to enhanced user satisfaction, offering important implications for MaaS service providers and urban transportation planners.

This study faces limitations, primarily the geographic homogeneity of the survey respondents, which may not account for national or regional variations. Additionally, certain influential factors on MaaS sustainability, such as broader service experience and user satisfaction elements, were not fully explored due to data constraints. Future research should address these limitations by incorporating a broader geographic scope and examining additional factors that influence MaaS sustainability. Real-world case studies could provide further depth to the analysis and support the development of more targeted and effective sustainable urban transportation strategies.

## Supporting information

S1 File Data. (DOCX)
