# Supplementary material for: Enhancing MaaS user satisfaction through strategic marketing: The synergy of sustainability and service experience
Source: PLoS One. 2025 Jan 27;20(1):e0316753. doi: 10.1371/journal.pone.0316753 (PMC11771868; doi:10.1371/journal.pone.0316753)
Supplement: S1 File — (DOCX) [file pone.0316753.s001.docx]

## **Analysis report**

## **1、Background information statistics**

A total of 450 questionnaires were collected in this survey, of which 33 samples were filled out with omissions, or the options were inconsistent or there was an obvious regularity in the answers, which were considered invalid samples. After eliminating the invalid samples, 417 valid questionnaires remained, and the questionnaire survey efficiency was 92.67%. The background information of the subjects: gender, age, education, and statistical results are shown in the table below.

**Distribution of samples**

|  | Categories | Frequency (times) | Percentage (%) |
| --- | --- | --- | --- |
| Gender | male | 188 | 45.1 |
|  | female | 229 | 54.9 |
| Age | Under 18 years old | 23 | 5.5 |
|  | 18-25 | 82 | 19.7 |
|  | 26-40 | 144 | 34.5 |
|  | 41-60 | 89 | 21.3 |
|  | Over 60 years old | 79 | 18.9 |
| Education | Junior high school and below | 25 | 6 |
|  | high school | 55 | 13.2 |
|  | university | 233 | 55.9 |
|  | Graduate or above | 104 | 24.9 |
| MaaS service usage | never used | 30 | 7.2 |
|  | 1 to 5 times a year | 255 | 61.2 |
|  | More than 5 times a year | 132 | 31.6 |

## **2、Descriptive Statistics**

The relationship model proposed in the study includes inner peace, critical moments, outcome focus, product experience, safety, economy, environment, society, Mass user satisfaction, and MaaS publicity, 10 variables, and 30 items. All scales use the Likert 5-point scale, with a maximum of 5 and a minimum of 1. For all variables, the higher the score, the higher the corresponding evaluation level. The absolute values ​​of skewness and kurtosis of all items are less than 2 and 7, indicating that the sample distribution meets the normality. The descriptive statistical results of the 30 measurement items are shown in the table.

Descriptive Statistics

| Variable Item | Question | Minimum | Maximum | mean value | Standard Deviation | Skewness | Kurtosis |
| --- | --- | --- | --- | --- | --- | --- | --- |
| peace of mind | PM1 | 1 | 5 | 3.58 | 1.323 | -0.567 | -0.894 |
|  | PM2 | 1 | 5 | 3.51 | 1.282 | -0.607 | -0.736 |
|  | PM3 | 1 | 5 | 3.54 | 1.282 | -0.596 | -0.771 |
| moments of truth | MT1 | 1 | 5 | 3.47 | 1.248 | -0.530 | -0.794 |
|  | MT2 | 1 | 5 | 3.51 | 1.296 | -0.500 | -0.887 |
|  | MT3 | 1 | 5 | 3.41 | 1.266 | -0.481 | -0.825 |
| outcome focus | OF1 | 1 | 5 | 3.56 | 1.194 | -0.544 | -0.619 |
|  | OF2 | 1 | 5 | 3.47 | 1.213 | -0.545 | -0.694 |
|  | OF3 | 1 | 5 | 3.47 | 1.164 | -0.421 | -0.783 |
| product experience | PE1 | 1 | 5 | 3.4 | 1.183 | -0.338 | -0.783 |
|  | PE2 | 1 | 5 | 3.49 | 1.125 | -0.431 | -0.700 |
|  | PE3 | 1 | 5 | 3.41 | 1.159 | -0.304 | -0.832 |
| safe | SA1 | 1 | 5 | 3.77 | 1.064 | -0.527 | -0.664 |
|  | SA2 | 1 | 5 | 3.67 | 1.029 | -0.481 | -0.347 |
|  | SA3 | 1 | 6 | 3.72 | 1.082 | -0.484 | -0.525 |
| economy | EC1 | 1 | 5 | 3.43 | 1.059 | -0.265 | -0.510 |
|  | EC2 | 1 | 5 | 3.43 | 1.109 | -0.202 | -0.869 |
|  | EC3 | 1 | 5 | 3.37 | 1.071 | -0.253 | -0.541 |
| environment | EN1 | 1 | 5 | 3.59 | 0.937 | -0.651 | 0.338 |
|  | EN2 | 1 | 5 | 3.53 | 0.958 | -0.414 | 0.019 |
|  | EN3 | 1 | 5 | 3.46 | 0.945 | -0.407 | 0.131 |
| society | SO1 | 1 | 5 | 3.68 | 1.036 | -0.569 | -0.157 |
|  | SO2 | 1 | 5 | 3.6 | 1.026 | -0.478 | -0.238 |
|  | SO3 | 1 | 5 | 3.59 | 0.980 | -0.448 | 0.021 |
| User satisfaction with MaaS | US1 | 1 | 5 | 3.42 | 1.242 | -0.272 | -1.119 |
|  | US2 | 1 | 5 | 3.41 | 1.234 | -0.444 | -0.863 |
|  | US3 | 1 | 5 | 3.33 | 1.284 | -0.408 | -0.966 |
| MaaS Advocacy | MA1 | 1 | 5 | 3.7 | 0.937 | -0.609 | 0.266 |
|  | MA2 | 1 | 5 | 3.94 | 1.025 | -0.973 | 0.642 |
|  | MA3 | 1 | 5 | 3.72 | 0.962 | -0.692 | 0.472 |

**3、Reliability test**

Reliability, also known as reliability, refers to the credibility of the questionnaire, which mainly reflects the consistency, consistency, reproducibility and stability of the test results. A good measurement tool should measure the same thing repeatedly, and its results should remain unchanged to be credible. This article uses the α coefficient to represent the consistency reliability within the scale. The higher the α value, the more consistent the results of the items in the questionnaire are, which means that the reliability of the scale is better. When the α coefficient is lower than 0.6, it is low reliability, and it is necessary to consider recompiling the questionnaire or screening controversial indicators in the questionnaire. Reliability higher than 0.9 indicates that the questionnaire data results are very stable, and 0.7 to 0.8 is relatively stable.

The above method is used to evaluate: the reliability range of each dimension of the questionnaire: inner peace, critical moments, result focus, product experience, safety, economy, environment, society, Mass user satisfaction, and MaaS promotion variables is 0.768-0.852. It can be seen from the data in the table that the results are highly stable and have a certain degree of credibility.

Reliability test of each variable

| Dimensions | Number of items | Cronbach's Alpha | Cronbach's Alpha based on standardized items |
| --- | --- | --- | --- |
| peace of mind | 3 | 0.83 | 0.830 |
| moments of truth | 3 | 0.831 | 0.831 |
| outcome focus | 3 | 0.768 | 0.768 |
| product experience | 3 | 0.801 | 0.801 |
| safe | 3 | 0.824 | 0.824 |
| economy | 3 | 0.852 | 0.852 |
| environment | 3 | 0.839 | 0.839 |
| society | 3 | 0.821 | 0.821 |
| User satisfaction with MaaS | 3 | 0.839 | 0.840 |
| MaaS Advocacy | 3 | 0.832 | 0.833 |

1. **Validity Analysis (Confirmatory Factor Analysis)**

Main evaluation indicators and evaluation criteria for the overall fitness of the model

| **Indicators** | **Value range** | **Ideal value** |
| --- | --- | --- |
| X^2^/df | Greater than 0 | Less than 5, less than 3 is better |
| RMSEA | Greater than 0 | Less than 0.1, the fit is good; less than 0.08, the fit is very good; less than |
|  |  | 0.05, the fit is very good; below 0.01, the fit is excellent |
| GFI | between 0~1 | Greater than 0.8 is acceptable; greater than 0.9 is best |
| CFI | between 0~1 | Greater than 0.8 is acceptable; greater than 0.9 is best |
| IFI | between 0~1 | Greater than 0.8 is acceptable; greater than 0.9 is best |
| TFI | between 0~1 | Greater than 0.8 is acceptable; greater than 0.9 is best |
| AGFI | between 0~1 | Greater than 0.8 is acceptable; greater than 0.9 is best |

**1、Sustainable**


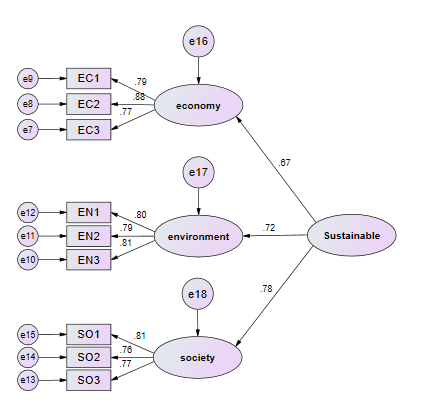


Sustainable

Sustainable model fit indicators

| Indicator | χ^2^/df | GFI | AGFI | IFI | TLI | CFI | RMSEA |
| --- | --- | --- | --- | --- | --- | --- | --- |
| Statistical value | 2.301 | 0.972 | 0.947 | 0.982 | 0.973 | 0.982 | 0.056 |
| Reference value | <5 | >0.8 | >0.8 | >0.8 | >0.8 | >0.8 | <0.08 |
| Standard achievement | Meet standard | Meet standard | Meet standard | Meet standard | Meet standard | Meet standard | Meet standard |

The model running fitting indicators are shown in the above table, and the fitting indicators are: χ^2^/df=2.301，GFI=0.972，AGFI=0.947，IFI=0.982，TLI=0.973，CFI=0.982，RMSEA=0.056，According to the fitting criteria of the comparison table, the fitting indices of the model of confirmatory factor analysis all meet the requirements and are suitable for model analysis.

**Convergent validity test**

Convergent validity, also known as convergent validity, means that test indicators measuring the same latent traits (concepts) will fall on the same common factor: Convergent validity is tested through construct reliability (CR) and average variance extracted (AVE). The combined reliability is usually greater than 0.7 and the AVE is greater than 0.5, which is the reference value to reach the standard.

The results of sustainable convergent validity analysis

| Variables | Item | b | SE | C.R | P | β | CR | AVE |
| --- | --- | --- | --- | --- | --- | --- | --- | --- |
| Sustainable | economy | 1 |  |  |  | 0.672 | 0.769 | 0.527 |
|  | environment | 1.001 | 0.129 | 7.748 | *** | 0.722 |  |  |
|  | society | 1.064 | 0.141 | 7.556 | *** | 0.779 |  |  |
| economy | EC3 | 1 |  |  |  | 0.767 | 0.854 | 0.662 |
|  | EC2 | 1.186 | 0.07 | 16.883 | *** | 0.878 |  |  |
|  | EC1 | 1.019 | 0.064 | 15.917 | *** | 0.791 |  |  |
| environment | EN3 | 1 |  |  |  | 0.810 | 0.840 | 0.636 |
|  | EN2 | 0.985 | 0.062 | 15.834 | *** | 0.787 |  |  |
|  | EN1 | 0.974 | 0.061 | 15.975 | *** | 0.796 |  |  |
| society | SO3 | 1 |  |  |  | 0.770 | 0.822 | 0.606 |
|  | SO2 | 1.031 | 0.072 | 14.346 | *** | 0.758 |  |  |
|  | SO1 | 1.109 | 0.074 | 14.934 | *** | 0.807 |  |  |

Sustainable: The standardized factor loadings, combined reliability (C.R.) and average variance extracted (AVE) of the observed values of Economy, environment, and society are shown in the table. The factor loading values of each item are all greater than 0.6, indicating that the convergent validity is high, the composite reliability (C.R.) of each dimension is greater than 0.7 and meets the standard, and the AVE is greater than 0.5 and meets the standard, indicating that the scale of this study has good convergent validity.

**Discriminant validity**

Discriminant validity: A test has discriminant validity if it can be statistically proven that indicators that are not expected to be related to the presumed construct are not related to the construct.

Sustainable Discriminant Validity Analysis

|  | society | environment | economy |
| --- | --- | --- | --- |
| society | 0.779 |  |  |
| environment | 0.562 | 0.798 |  |
| economy | 0.523 | 0.485 | 0.813 |

As can be seen from the table above, the square root of the AVE of each dimension of Economy, Environment, and Society is greater than the correlation coefficient between each dimension, so it shows that the scale has good discriminant validity.

**2、MaaS User Experience**


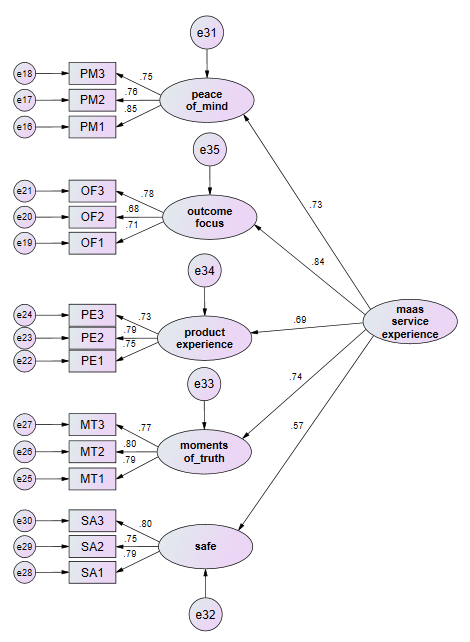


MaaS User Experience

Model fit indicators of MaaS user experience

| Indicator | χ2/df | GFI | AGFI | IFI | TLI | CFI | RMSEA |
| --- | --- | --- | --- | --- | --- | --- | --- |
| Statistical value | 1.570 | 0.961 | 0.945 | 0.982 | 0.977 | 0.982 | 0.037 |
| Reference value | <5 | >0.8 | >0.8 | >0.8 | >0.8 | >0.8 | <0.08 |
| Standard achievement | Meet standard | Meet standard | Meet standard | Meet standard | Meet standard | Meet standard | Meet standard |

The model running fitting indicators are shown in the above table, and the fitting indicators are: χ2/df=1.570，GFI=0.961，AGFI=0.945，IFI=0.982，TLI=0.977，CFI=0.982，RMSEA=0.037，According to the fitting criteria of the comparison table, the fitting indices of the model of confirmatory factor analysis all meet the requirements and are suitable for model analysis.

**Convergent validity test**

Convergent validity, also known as convergent validity, means that test indicators measuring the same latent traits (concepts) will fall on the same common factor: Convergent validity is tested through construct reliability (CR) and average variance extracted (AVE). The combined reliability is usually greater than 0.7 and the AVE is greater than 0.5, which is the reference value to reach the standard.

Results of convergent validity analysis of MaaS user experience

| Variables | Item | b | SE | C.R | P | β | CR | AVE |
| --- | --- | --- | --- | --- | --- | --- | --- | --- |
| MaaS service experience | peace of mind | 1 |  |  |  | 0.729 | 0.841 | 0.517 |
|  | outcome focus | 0.864 | 0.09 | 9.552 | *** | 0.838 |  |  |
|  | product experience | 0.752 | 0.083 | 9.004 | *** | 0.692 |  |  |
|  | moments of truth | 0.893 | 0.092 | 9.683 | *** | 0.738 |  |  |
|  | safe | 0.591 | 0.072 | 8.156 | *** | 0.574 |  |  |
| peace of mind | PM1 | 1 |  |  |  | 0.848 | 0.831 | 0.621 |
|  | PM2 | 0.868 | 0.055 | 15.754 | *** | 0.760 |  |  |
|  | PM3 | 0.86 | 0.055 | 15.629 | *** | 0.753 |  |  |
| Outcome focus | OF1 | 1 |  |  |  | 0.705 | 0.767 | 0.524 |
|  | OF2 | 0.977 | 0.083 | 11.754 | *** | 0.679 |  |  |
|  | OF3 | 1.083 | 0.084 | 12.92 | *** | 0.783 |  |  |
| product experience | PE1 | 1 |  |  |  | 0.751 | 0.802 | 0.575 |
|  | PE2 | 1.005 | 0.072 | 13.9 | *** | 0.794 |  |  |
|  | PE3 | 0.95 | 0.072 | 13.211 | *** | 0.728 |  |  |
| moments of truth | MT1 | 1 |  |  |  | 0.793 | 0.831 | 0.622 |
|  | MT2 | 1.049 | 0.067 | 15.723 | *** | 0.801 |  |  |
|  | MT3 | 0.987 | 0.065 | 15.259 | *** | 0.771 |  |  |
| safe | SA1 | 1 |  |  |  | 0.790 | 0.825 | 0.610 |
|  | SA2 | 0.92 | 0.064 | 14.392 | *** | 0.752 |  |  |
|  | SA3 | 1.03 | 0.069 | 14.946 | *** | 0.801 |  |  |

MaaS User Experience Scale: Inner Peace, Moments of Truth, Result Focus, Product Experience, Safety Dimensions Observed Value Factor Loading, Combined Reliability (C.R.) and Average Variance Extracted (AVE) are shown in the table. The factor loading values of each item are all greater than 0.6, indicating that the convergent validity is high, the combined reliability (C.R.) of each dimension is greater than 0.7 and meets the standard, and the AVE is greater than 0.5 and meets the standard, indicating that the scale of this study has good convergent validity.

**Discriminant validity**

Discriminant validity: A test has discriminant validity if it can be statistically proven that indicators that are not expected to be related to the presumed construct are not related to the construct.

Discriminant validity analysis of MaaS user experience

|  | safe | moments of truth | product experience | outcome focus | peace of mind |
| --- | --- | --- | --- | --- | --- |
| safe | 0.781 |  |  |  |  |
| moments of truth | 0.424 | 0.788 |  |  |  |
| product experience | 0.397 | 0.511 | 0.758 |  |  |
| outcome focus | 0.482 | 0.619 | 0.580 | 0.724 |  |
| peace of mind | 0.418 | 0.538 | 0.504 | 0.611 | 0.788 |

From the above table, we can see that the square root of AVE of each dimension of social sustainability: inner peace, critical moments, result focus, product experience, and safety is greater than the correlation coefficient between the dimensions, which means that the scale has good discriminant validity.

**3、Overall scale**


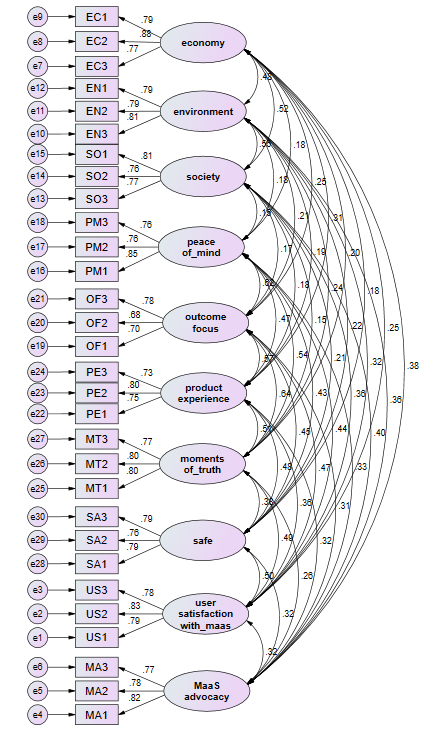


Overall scale

Model fit index for the overall scale

| Indicator | χ2/df | GFI | AGFI | IFI | TLI | CFI | RMSEA |
| --- | --- | --- | --- | --- | --- | --- | --- |
| Statistical value | 1.187 | 0.938 | 0.921 | 0.988 | 0.986 | 0.988 | 0.021 |
| Reference value | <5 | >0.8 | >0.8 | >0.8 | >0.8 | >0.8 | <0.08 |
| Standard achievement | Meet standard | Meet standard | Meet standard | Meet standard | Meet standard | Meet standard | Meet standard |

The model running fitting indicators are shown in the above table, and the fitting indicators are: χ2/df=1.187，GFI=0.938，AGFI=0.921，IFI=0.988，TLI=0.986，CFI=0.988，RMSEA=0.021，According to the fitting criteria of the comparison table, the fitting indices of the model of confirmatory factor analysis all meet the requirements and are suitable for model analysis.

**Convergent validity test**

Convergent validity, also known as convergent validity, means that test indicators measuring the same latent traits (concepts) will fall on the same common factor: Convergent validity is tested through construct reliability (CR) and average variance extracted (AVE). The combined reliability is usually greater than 0.7 and the AVE is greater than 0.5, which is the reference value to reach the standard.

Results of convergent validity analysis of the overall scale

| Variables | Item | b | SE | C.R | P | β | CR | AVE |
| --- | --- | --- | --- | --- | --- | --- | --- | --- |
| user satisfaction with maas | US1 | 1 |  |  |  | 0.785 | 0.840 | 0.637 |
|  | US2 | 1.051 | 0.064 | 16.315 | *** | 0.830 |  |  |
|  | US3 | 1.025 | 0.066 | 15.555 | *** | 0.778 |  |  |
| MaaS advocacy | MA1 | 1 |  |  |  | 0.822 | 0.834 | 0.626 |
|  | MA2 | 1.041 | 0.067 | 15.516 | *** | 0.781 |  |  |
|  | MA3 | 0.962 | 0.063 | 15.348 | *** | 0.770 |  |  |
| economy | EC3 | 1 |  |  |  | 0.769 | 0.854 | 0.662 |
|  | EC2 | 1.183 | 0.069 | 17.079 | *** | 0.878 |  |  |
|  | EC1 | 1.014 | 0.063 | 15.976 | *** | 0.789 |  |  |
| environment | EN3 | 1 |  |  |  | 0.810 | 0.840 | 0.636 |
|  | EN2 | 0.986 | 0.062 | 15.946 | *** | 0.788 |  |  |
|  | EN1 | 0.971 | 0.061 | 16.039 | *** | 0.794 |  |  |
| society | SO3 | 1 |  |  |  | 0.772 | 0.822 | 0.606 |
|  | SO2 | 1.027 | 0.071 | 14.467 | *** | 0.756 |  |  |
|  | SO1 | 1.105 | 0.073 | 15.128 | *** | 0.806 |  |  |
| peace of mind | PM1 | 1 |  |  |  | 0.848 | 0.831 | 0.621 |
|  | PM2 | 0.867 | 0.055 | 15.793 | *** | 0.758 |  |  |
|  | PM3 | 0.864 | 0.055 | 15.753 | *** | 0.756 |  |  |
| outcome focus | OF1 | 1 |  |  |  | 0.700 | 0.767 | 0.524 |
|  | OF2 | 0.99 | 0.084 | 11.786 | *** | 0.682 |  |  |
|  | OF3 | 1.092 | 0.084 | 12.925 | *** | 0.784 |  |  |
| product experience | PE1 | 1 |  |  |  | 0.749 | 0.802 | 0.575 |
|  | PE2 | 1.01 | 0.072 | 14.053 | *** | 0.796 |  |  |
|  | PE3 | 0.952 | 0.072 | 13.281 | *** | 0.728 |  |  |
| moments of truth | MT1 | 1 |  |  |  | 0.796 | 0.831 | 0.621 |
|  | MT2 | 1.04 | 0.066 | 15.866 | *** | 0.798 |  |  |
|  | MT3 | 0.981 | 0.064 | 15.398 | *** | 0.770 |  |  |
| safe | SA1 | 1 |  |  |  | 0.792 | 0.825 | 0.611 |
|  | SA2 | 0.928 | 0.063 | 14.714 | *** | 0.760 |  |  |
|  | SA3 | 1.016 | 0.067 | 15.14 | *** | 0.792 |  |  |

The overall scale scale: peace of mind、outcome focus、moments of truth、product experience、safe、economy,、environment、 society、Mass user satisfaction,、MaaS publicity, the observed value factor loading, combined reliability (C.R.) and average variance extracted (AVE) of each dimension are shown in the table. The factor loading value of each item is greater than 0.6, indicating that the convergent validity is high, the combined reliability (C.R.) of each dimension is greater than 0.7 and meets the standard, and the AVE is greater than 0.5 and meets the standard, indicating that the scale of this study has good convergent validity.

**Discriminant validity**

Discriminant validity: If a test can be statistically proven to have no correlation with the pre-determined construct, then the test has discriminant validity.

Discriminant validity analysis of the overall scale

|  | **1** | **2** | **3** | **4** | **5** | **6** | **7** | **8** | **9** | **10** |
| --- | --- | --- | --- | --- | --- | --- | --- | --- | --- | --- |
| user satisfaction with maas | **0.798** |  |  |  |  |  |  |  |  |  |
| economy | 0.251 | **0.813** |  |  |  |  |  |  |  |  |
| MaaS advocacy | 0.317 | 0.376 | **0.791** |  |  |  |  |  |  |  |
| environment | 0.317 | 0.485 | 0.357 | **0.797** |  |  |  |  |  |  |
| society | 0.360 | 0.523 | 0.400 | 0.562 | **0.778** |  |  |  |  |  |
| peace of mind | 0.438 | 0.181 | 0.330 | 0.180 | 0.150 | **0.788** |  |  |  |  |
| outcome focus | 0.468 | 0.247 | 0.312 | 0.210 | 0.170 | 0.620 | **0.724** |  |  |  |
| product experience | 0.362 | 0.306 | 0.321 | 0.193 | 0.184 | 0.474 | 0.569 | **0.758** |  |  |
| moments of truth | 0.491 | 0.204 | 0.261 | 0.243 | 0.151 | 0.541 | 0.640 | 0.506 | **0.788** |  |
| safe | 0.495 | 0.178 | 0.318 | 0.224 | 0.210 | 0.434 | 0.447 | 0.484 | 0.384 | **0.781** |

From the above table, we can see that the overall scale:peace of mind、outcome focus、moments of truth、product experience、safe、economy、environment、society、Mass user satisfaction, MaaS publicity, The square root of AVE of each dimension is greater than the correlation coefficient between the dimensions, which indicates that the scale has good discriminant validity.

**5、Structural equation model fit test**

The main path results of the model are shown in the figure below. According to the structural equation model fitness test indicators: the ratio of the chi-square degrees of freedom should be between 1 and 3, and less than 5 is acceptable; the asymptotic residual mean square error RMSEA (Residual mean square error of approximation) value should be between 0.05 and 0.08. If it is less than 0.05, it means that the fit is very good; the goodness of fit index GFI value is generally considered to be greater than 0.9, if it is above 0.8, it is acceptable; the value-added fitness index CFI value should be greater than 0.9; the non-standard fitness index TLI value should be above 0.9; it is generally believed that the sample size should be greater than 200.

Main evaluation indicators and evaluation criteria for the overall fitness of the model

| Indicators | Range of values | Ideal value |
| --- | --- | --- |
| χ²/df | Greater than 0 | Less than 5, less than 3 is better |
| RMSEA | Greater than 0 | Less than 0.1, good fit; less than 0.08, very good fit; less than |
|  |  | 0.05, very good fit; less than 0.01, excellent fit |
| GFI | between 0~1 | Greater than 0.8 is acceptable; greater than 0.9 is the best |
| CFI | between 0~1 | Greater than 0.8 is acceptable; greater than 0.9 is the best |
| IFI | between 0~1 | Greater than 0.8 is acceptable; greater than 0.9 is the best |
| TFI | between 0~1 | Greater than 0.8 is acceptable; greater than 0.9 is the best |
| AGFI | between 0~1 | Greater than 0.8 is acceptable; greater than 0.9 is the best |


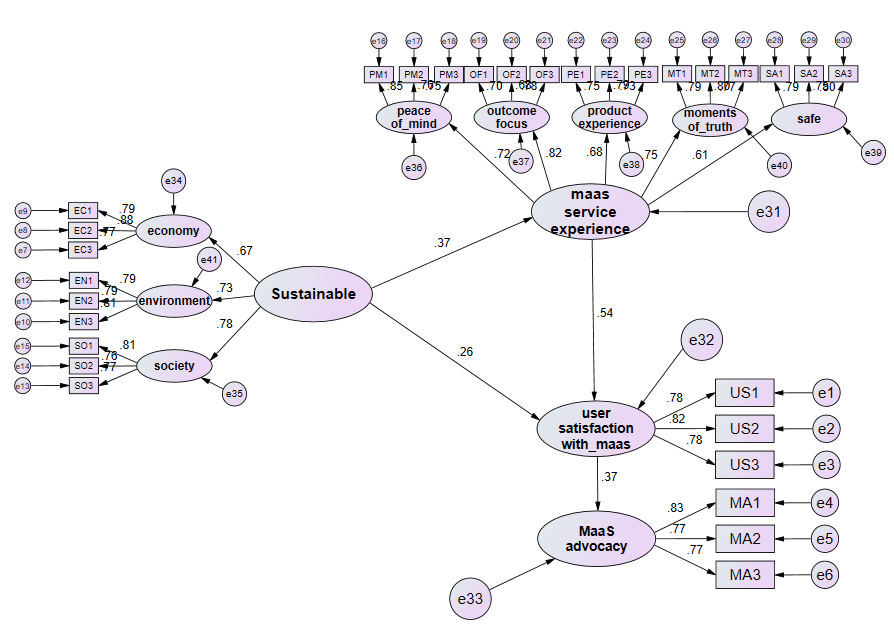


Structural equation model diagram operation results (standardized)

Structural equation model fit index (standardized)

| Indicator | χ2/df | GFI | AGFI | IFI | TLI | CFI | RMSEA |
| --- | --- | --- | --- | --- | --- | --- | --- |
| Statistical value | 1.368 | 0.923 | 0.909 | 0.975 | 0.972 | 0.974 | 0.030 |
| Reference value | <5 | >0.8 | >0.8 | >0.8 | >0.8 | >0.8 | <0.08 |
| Standard achievement | Meet standard | Meet standard | Meet standard | Meet standard | Meet standard | Meet standard | Meet standard |

The fit indicators of the equation model are shown in the table above: χ²/df=1.368，；RMSEA=0.030；GFI=0.923、AGFI=0.909、CFI=0.991、IFI=0.991、TLI=0.988；According to the fitting criteria of the comparison table, the fitting indicators of the structural equation model all meet the requirements and are suitable for structural equation model analysis.

**Path analysis**

This study uses AMOS26.0 software to perform path analysis of the structural equation model, thereby obtaining the path coefficient value and C.R. value of the structural equation model. The path coefficient reflects the influence relationship and degree between variables. The critical ratio C.R. (Critical Ratio) can determine whether the regression coefficient is significant or not. It is generally believed that a C.R. value greater than or equal to 1.96 indicates a significant difference at the 0.05 significance level. The standardized regression coefficient and variance parameter estimation of the structural equation model in this study are shown in the table.

The path coefficients between the variables

| Regression Path | | | β | b | S.E. | C.R. | P |
| --- | --- | --- | --- | --- | --- | --- | --- |
| MaaS service experience | ← | Sustainable | 0.373 | 0.551 | 0.111 | 4.981 | *** |
| user satisfaction with MaaS | ← | Sustainable | 0.256 | 0.451 | 0.113 | 3.988 | *** |
| user satisfaction with MaaS | ← | maas service experience | 0.541 | 0.645 | 0.083 | 7.733 | *** |
| MaaS advocacy | ← | user satisfaction with maas | 0.365 | 0.293 | 0.047 | 6.252 | *** |

Notes: ***, P < 0.001; b: unstandardized coefficient; β: standardized factor coefficient

1、Sustainable has a significant positive impact on maas service experience

The data analysis results are: after standardization, the coefficient of its path is 0.373, and the CR value, i.e. the critical ratio value, is 4.981, which is greater than 1.96, and the corresponding P < 0.001. Combining the above indicators, it can be seen that the path described in the hypothesis is significant, proving that the hypothesis is valid.

2、Sustainable has a significant positive impact on user satisfaction with MAAS

The data analysis results are: after standardization, the coefficient of its path is 0.256, and the CR value, i.e. the critical ratio value, is 3.988, which is greater than 1.96, and the corresponding P < 0.001. Combining the above indicators, it can be seen that the path described in the hypothesis is significant, proving that the hypothesis is valid.

3、Maas service experience has a significant positive impact on user satisfaction with Maas

The data analysis results are: after standardization, the coefficient of its path is 0.541, and the CR value, that is, the critical ratio value, is 7.733, which is greater than 1.96, and the corresponding P is less than 0.001. Combining the above indicators, it can be seen that the path described in the hypothesis is significant, proving that the hypothesis is valid.

4、User satisfaction with maas has a significant positive impact on MaaS advocacy

The data analysis results are: after standardization, the coefficient of the path is 0.365, and the CR value, i.e. the critical ratio value, is 6.252, which is greater than 1.96, and the corresponding P < 0.001. Combining the above indicators, it can be seen that the path described in the hypothesis is significant, proving that the hypothesis is valid.

**6、Mediation effect test**

The existence of mediation effect can be directly tested by Bootstrap method. The assumption of direct test is H0: ab=0. If the confidence interval of the test result contains 0, it means that there is no mediation effect.

According to the results of path analysis, the hypothesis test is valid. In order to explore whether there is a mediation effect in these significant paths, we run Bootstrap method in AMOS26.0, choose to repeat 5000 times, the confidence interval standard is 95%, and the bias correction method is used for testing.

Mediating Effect

| Regression Path | Effect size | SE | 95% lower limit | 95% upper limit | P |
| --- | --- | --- | --- | --- | --- |
| Sustainable→maas service experience→user satisfaction with maas | 0.202 | 0.045 | 0.124 | 0.302 | 0.000 |
| Sustainable→user satisfaction with maas→MaaS advocacy | 0.198 | 0.042 | 0.123 | 0.284 | 0.000 |
| maas service experience→user satisfaction with maas→MaaS advocacy | 0.093 | 0.030 | 0.043 | 0.160 | 0.000 |

The bootstrap method is used to analyze the mediating effect of maas service experience between Sustainable and user satisfaction with maas

user satisfaction with maas between Sustainable and MaaS advocacy, and the mediating effect of user satisfaction with maas between maas service experience and MaaS advocacy. The results are shown in the following table.

The bias-corrected confidence interval of the bootstap of Sustainable→maas service experience→user satisfaction with maas is [0.124, 0.302] and does not include 0, indicating that the mediating effect is established.

The bias-corrected confidence interval of the bootstap of Sustainable→user satisfaction with maas→MaaS advocacy is [0.123, 0.284] and does not include 0, indicating that the mediating effect is established.

The bias-corrected confidence interval of the bootstap of maas service experience→user satisfaction with maas→MaaS advocacy is [0.043, 0.160] and does not include 0, indicating that the mediating effect is established.
